# Low birth weight contributed to increased serum IL-6 levels in infantile respiratory syncytial virus infection

**DOI:** 10.1186/s12887-017-0961-2

**Published:** 2017-12-16

**Authors:** Yuan-Jian Sheng, Shan-Shan Xu, Xue-Jing Li, Jin-Ling Liu, Xi-Ling Wu, Xue-Feng Xu

**Affiliations:** 1 Department of Respiratory Medicine, The Children’s Hospital, Zhejiang University School of Medicine, Hangzhou, 310052 China; 2 Department of Neonatology, The Children’s Hospital, Zhejiang University School of Medicine, Hangzhou, 310052 People’s Republic of China

**Keywords:** Cytokine, Infant, Lower respiratory tract infection, Low birth weight, Respiratory syncytial virus

## Abstract

**Background:**

To evaluate the role of serum cytokines in the pathogenesis of respiratory syncytial virus (RSV) infection in infants with low birth weight (LBW).

**Methods:**

A prospective observational study was performed, and hospitalized children with lower respiratory tract infection (LRTI) were recruited. Three hundred fifty-eight patients < 1 year met the inclusion criteria: 116 patients had only RSV infection (RSV group); 242 patients had no RSV or other specific pathogen (non-RSV group). Serum interleukin-2 (IL-2), IL-4, IL-6, IL-10, tumor necrosis factor-α (TNF-α), and interferon-γ (IFN-γ) were detected through flow cytometry.

**Results:**

No significant differences in serum IL-2, 4, 6, 10, and IFN-γ levels were observed between the RSV and non-RSV groups. For RSV infected infants with or without wheezing, delivery mode had no obvious effect on the changes of serum cytokine levels. However, the level of IL-6 in the RSV-infected infants with LBW was significantly higher than that in infants with normal birth weight.

**Conclusions:**

Serum IL-6 level was significantly increased in RSV infected infants with LBW. It is likely that the specific serum cytokine pattern will contribute to our understanding of the pathogenesis of RSV infections, especially in RSV-infected infants with LBW.

## Background

Lower respiratory tract infection (LRTI) is the leading cause of global child mortality. Respiratory syncytial virus (RSV) is believed to be the most important viral pathogen causing LRTI in young children. Furthermore, the incidence for severe LRTI was highest in infants aged 0–11 months [1]. RSV is not only the leading pathogen of LRTI including pneumonia and bronchiolitis, but is also closely associated with the development of asthma [2]. A number of clinical epidemiology studies have revealed that there is a strong association between RSV bronchiolitis in infancy and the development of wheezing or asthma in later childhood. About 40% of children after severe RSV bronchiolitis subsequently develop asthma [2, 3]. Despite the importance of RSV as a respiratory pathogen, the underlying mechanisms of the development of subsequent wheezing and asthma following primary RSV infection remain unclear.

RSV infection may increase the susceptibility to developing allergic immune responses by breaking immune tolerance to allergens early in life through regulatory T cells [4]. Moreover, RSV-induced neurogenic inflammation appears to potentiate neural pathways that favor bronchoconstriction and mucus production [5]. Interestingly, studies have shown that infection with RSV also results in a significant increase in proinflammatory cytokines such as interleukin (IL)-1, IL-6, IL-8, interferon (IFN)-γ, and tumor necrosis factor (TNF)-α, contributing to development of recurrent wheezing or asthma [3]. Our previous study has shown that low birth weight (LBW) or intrauterine growth retardation significantly increases the risk of childhood asthma. Children with LBW have an approximately 16% higher risk of asthma compared with those with normal birth weight (NBW) [6]. Another study by Rossi et al. further revealed that LBW was not only associated with the increased risk of asthma or wheezing, but also with RSV infection severity [7].

Although we recognize the significant role of low birth weight in infantile RSV-induced LRTI or wheezing, the mechanism of this phenomenon remains unknown. In view of the potential role of cytokines, we hypothesized that LBW induces a specific cytokine profile in infantile bronchiolitis or wheezing induced by RSV infection, leading to more significant manifestations related to asthma. The present study aims to evaluate the possible role of cytokines in the pathogenesis of RSV infection in infants with LBW.

## Methods

### Data collection

In this prospective observational study, hospitalized children with lower respiratory tract infections (LRTI) from the Respiratory Department of the Children’s Hospital of Zhejiang University School of Medicine between January 2013 and December 2014 were recruited. LRTI, including bronchiolitis and pneumonia, has been defined as equivalent to clinical pneumonia, which is characterized by acute onset cough or difficulty breathing with fast breathing for age [8]. Inclusion criteria were hospital admission, LRTI, age <1 year and positive respiratory immunofluorescence detection for RSV [9]. Patients with severe illness and non-severe illness were distinguished by the need for intensive care. Wheezing is defined as a continuous high-pitched sound with musical quality emitting from the chest during expiration [10]. Wheezing symptoms were assessed by at least two clinicians from our study team or the treating physicians (during the weekend). Records on admission age, birth weight, gender, diagnosis, viral pathogen detection, bacterial culture results, laboratory test, length of stay and other relevant medical conditions were collected. Those patients with chronic lung diseases, congenital heart disease, cerebral palsy or tumor were excluded.

### Identification of respiratory virus in nasopharyngeal aspirates (NPA)

Samples of nasopharyngeal aspirates (NPA) were obtained from the nasopharynx through catheters after instilling 5 ml of sterile physiologic saline solution into mucus traps at the time of hospitalization. Respiratory immunofluorescence detection via NPA for respiratory syncytial virus (RSV), influenza virus A and B, parainfluenza virus, and adenovirus were carried out. Serum at the time of admission was collected for *M. pneumoniae* antibody measurements: IgM and IgG to *M. pneumoniae* were tested by means of an enzyme-linked immunosorbent assay (ELISA) according to the manufacturer’s instructions. *M. pneumoniae* DNA in NPA specimens was detected by fluorescence quantitative real-time PCR. Meanwhile, sputum cultures were also performed.

### Cytokine measurement

Peripheral blood samples were collected at the time of admission. The blood sample was transferred to a serum separating tube and centrifuged at 1000 g at room temperature for 20 min after clotting. The serum was carefully harvested and the measurement of the cytokines was performed by flow cytometry immediately, or the serum was temporarily stored at 2–8 °C until analysis. Concentrations of IL-2, IL-4, IL-6, IL-10, TNF-α, and IFN-γ were quantitatively determined with use of the cytometric bead array (CBA) kit (CBA Human Th1/Th2 Cytokine Kit II; BD Biosciences, San Jose, California) as described previously [11]. The minimal and maximum limits of detection for all cytokines were 1 and 5000 pg/mL, respectively.

### Statistics

Statistical analysis was performed using descriptive statistics such as frequency and percentage, mean and standard error of mean (SEM). The continuous variables between two groups were compared by Student’s t-test. A general linear model was used to assess multiple potential confounders. All data were analyzed with PASW Statistics 18 software (SPSS Inc. Chicago, US). A *P*-value of less than 0.05 was considered to be statistically significant.

## Results

### Clinical characteristics

In the present study, 358 children with LRTI < 1 year met the inclusion criteria; 116 patients had only RSV infection (RSV group); 242 patients had no RSV or other specific pathogen infections (non-RSV group). No significant differences in birth weight, days of hospitalization, WBC counts, percentage of neutrophils and eosinophils were observed between the two groups of patients (Table 1). There were also no significant differences in birth weight, days of hospitalization, WBC counts, percentage of neutrophils and eosinophils between LBW (birth weight < 3.0 kg) and NBW (birth weight ≥ 3.0 kg) groups (Table 1). The mean age of the RSV group was significantly lower than that of the non-RSV group (Table 1). In RSV patients, the age of the LBW group was also significantly lower than that of the NBW group (Table 1). The values of C reactive protein (CRP) and procalcitonin (PCT) in both groups were within the normal reference ranges.

**Table 1:** Clinical characteristics of patients

Groups	Age (year)	Birth weight (kg)	Days of hospitalization	WBC (10^9/L)	N%	EOS%	CRP (mg/L)	PCT (ng/L)
Pathogens								
RSV (116)	0.29 ± 0.02**	3.24 ± 0.05	7.06 ± 0.47	10.22 ± 0.89	29.52 ± 1.42	1.74 ± 0.14	4.93 ± 0.97	0.16 ± 0.02
non-RSV (242)	0.60 ± 0.02	3.29 ± 0.03	8.69 ± 0.35	9.19 ± 0.17	33.78 ± 1.10	2.19 ± 0.17	4.96 ± 0.46	0.11 ± 0.01
Birth weight								
LBW (89)	0.50 ± 0.04	–	7.66 ± 0.58	9.20 ± 0.27	34.61 ± 1.93	2.00 ± 0.20	5.69 ± 0.79	0.13 ± 0.02
NBW (269)	0.50 ± 0.02	–	8.32 ± 0.32	9.63 ± 0.40	31.67 ± 0.98	2.01 ± 0.15	4.71 ± 0.52	0.12 ± 0.01
RSV patients								
LBW (32)	0.21 ± 0.03**	–	6.72 ± 0.91	8.56 ± 0.42	29.25 ± 1.90	2.03 ± 0.27	4.72 ± 1.06	0.16 ± 0.04
NBW (84)	0.33 ± 0.03	–	7.19 ± 0.56	10.85 ± 1.22	29.62 ± 1.82	1.63 ± 0.17	5.01 ± 1.23	0.16 ± 0.03

Continuous variables are represented by mean ± standard error of mean (SEM). **P < 0.01 for comparison with non-RSV group or NBW group. *WBC* white blood cell, *EOS* eosinophils, *CRP* C reactive protein, *PCT* procalcitonin, *RSV* respiratory syncytial virus, *LBW* Low birth weight, *NBW* normal birth weight

### Serum cytokine responses in relation to LRTI

In these patients with LRTI, serum IL-2 and IFN-γ levels in both the RSV and non-RSV groups were within the normal reference ranges (shown in Table 2). Although serum IL-4 and IL-10 levels were increased, there were no significantly statistical differences between the two groups. Serum IL-6 levels in the RSV group were similar to those in the non-RSV group. Compared with the increased average serum TNF-α level in the non-RSV group, average serum TNF-α level of the RSV group was within the normal reference range. A significantly statistical difference was observed between the groups (*P* = 0.024).

**Table 2:** Serum cytokine levels (pg/mL) among various groups

	No.	IL-2 (SEM)	IL-4 (SEM)	IL-6 (SEM)	IL-10 (SEM)	TNF-α (SEM)	IFN-γ (SEM)
Reference value		1.1–9.8	0.1–3.0	1.7–16.6	2.6–4.9	0.1–5.2	1.6–17.3
RSV group	116	3.98 (0.15)	3.25 (0.07)	54.06 (12.89)	9.87 (1.06)	*3.86 (1.24)	11.85 (1.08)
non-RSV group	242	3.51 (0.10)	3.31 (0.06)	60.30 (9.04)	8.21 (0.69)	9.81 (1.79)	15.54 (1.54)

In all subjects, *P = 0.024, as compared with non-RSV group. *RSV* respiratory syncytial virus, *IL* interleukin, *SEM* standard error of mean

### Serum cytokine levels in RSV patients

Serum cytokine levels of RSV-infected patients in different subgroups are shown in Table 3. Compared with RSV-infected infants with birth weight ≥ 3.0 kg (NBW), the level of IL-6 in RSV-infected infants with birth weight < 3.0 kg (LBW) was significantly increased. The general linear model revealed that body weight at admission, days of hospitalization, gender, delivery mode, and wheezing were not obviously associated with IL-6 levels. Only low birth weight (*P* = 0.035) was strongly correlated with changes of serum IL-6 levels. Although average serum TNF-α level in RSV-infected infants with LBW was higher than that in infants with NBW, there was no statistical difference between them. No significant differences in other cytokines were observed between birth weight subgroups. Likewise, for RSV infected infants, delivery mode and wheezing mode had no significant effects on the serum cytokine levels (Table 3).

**Table 3:** Serum cytokine levels (pg/mL) among RSV patients

	No.	IL-2 (SEM)	IL-4 (SEM)	IL-6 (SEM)	IL-10 (SEM)	TNF-α (SEM)	IFN-γ (SEM)
Reference value		1.1–9.8	0.1–3.0	1.7–16.6	2.6–4.9	0.1–5.2	1.6–17.3
Birth weight							
< 3.0 kg	32	3.90 (0.23)	3.53 (0.16)	*107.26 (34.23)	10.06 (1.23)	8.07 (4.44)	11.12 (1.03)
≥ 3.0 kg	84	4.01 (0.19)	3.14 (0.08)	33.79 (11.55)	9.79 (1.39)	2.26 0.12)	12.13 (1.45)
Delivery mode							
Vaginal delivery	64	3.99 (0.20)	3.31 (0.09)	67.66 (19.04)	9.01 (1.28)	3.06 (0.67)	11.85 (1.11)
Caesarean section	52	3.97 (0.23)	3.18 (0.11)	37.32 (16.59)	10.92 (1.75)	4.84 (2.64)	11.86 (2.00)
Wheezing							
No wheezing	31	3.83 (0.35)	3.04 (0.16)	42.49 (19.14)	11.94 (3.40)	2.33 (0.22)	10.22 (1.46)
With wheezing	85	4.03 (0.16)	3.32 (0.08)	58.28 (16.19)	9.12 (0.75)	4.41 (1.69)	12.45 (1.38)

*P < 0.05, as compared with RSV patients with birth weight ≥ 3.0 kg. *IL* interleukin, *SEM* standard error of mean

### Serum cytokine levels in RSV infants with wheezing

Serum cytokine levels of RSV-infected infants with wheezing in different subgroups are shown in Table 4. For RSV infected infants with wheezing, delivery mode had no obvious effect on the serum cytokine levels. Although average IL-6 level in the RSV-infected infants with wheezing and LBW was higher than that in infants with wheezing and NBW, there was no statistical difference between them. Average serum TNF-α level in the infants with wheezing and LBW was higher than that in infants with NBW, but there was also no statistical difference between them. No significant differences in other cytokines were observed between birth weight subgroups (Table 4).

**Table 4:** Serum cytokine levels (pg/mL) among RSV patients with wheezing

	No.	IL-2 (SEM)	IL-4 (SEM)	IL-6 (SEM)	IL-10 (SEM)	TNF-α (SEM)	IFN-γ (SEM)
Reference value		1.1–9.8	0.1–3.0	1.7–16.6	2.6–4.9	0.1–5.2	1.6–17.3
Birth weight							
< 3.0 kg	22	4.04 (0.26)	3.68 (0.19)	117.08 (44.04)	10.87 (1.48)	10.61 (6.43)	11.86 (1.35)
≥ 3.0 kg	63	4.03 (0.20)	3.20 (0.08)	37.75 (14.95)	8.50 (0.86)	2.25 (0.14)	12.66 (1.80)
Delivery mode							
Vaginal delivery	44	4.04 (0.20)	3.37 (0.11)	70.56 (24.55)	8.37 (1.03)	3.33 (0.97)	12.17 (1.28)
Caesarean section	41	4.03 (0.26)	3.27 (0.12)	45.10 (20.91)	9.91 (1.08)	5.58 (3.35)	12.75 (2.52)

*IL* interleukin, *SEM* standard error of mean

## Discussion

Respiratory syncytial virus (RSV) is the leading viral cause of acute LRTI worldwide, and the most important viral pathogen in infancy, especially among young infants less than 6 months [12]. A large number of studies have established a link between infantile RSV infection and wheezing or asthma [2]. Moreover, the increased evidence indicates that cytokines are strongly correlated with the pathogenic mechanism of RSV-induced wheezing or asthma attacks [13–17].

IL-6 is an important proinflammatory cytokine, promoting Th2 cell differentiation and simultaneously inhibiting Th1 cell polarization. TNF-α is another important proinflammatory cytokine that is involved in both acute and chronic inflammatory responses. IL-6 and TNF-α mRNA and protein levels in bronchoalveolar lavage fluid in term infants with RSV bronchiolitis were greater than the control group. However, in preterm infants with RSV bronchiolitis, IL-6 and TNF-α proteins significantly decreased [15]. The decrease in IL-6 and TNF-α protein levels in preterm infants may be correlated with the prolonged clinical course seen in those RSV patients [15]. Another study by Oda et al. demonstrated that serum IL-6 levels from patients with RSV infection were high compared with a control group, but there was no statistical significance [18]. Unlike local cytokine expression, our study indicated that systemic IL-6 levels in the RSV group were also high (greater than normal reference value), but there was no statistical significance compared with the non-RSV group. However, average serum TNF-α level from the RSV group was significantly lower than from the non-RSV group. It is likely that the decreased local or systemic TNF-α levels contributed to the prolonged clinical course and wheezing seen in those infants with RSV infection. Similar to other studies [13, 19, 20], no obvious changes in serum IL-2, 4, 10 and IFN-γ levels were observed in the present study.

A large number of studies have confirmed a link between infantile RSV infection and the development of wheezing or asthma in later childhood [2]. Moreover, individuals with LBW were more inclined to develop severe RSV infection, resulting in an increased risk of wheezing or asthma [6, 7]. The potential mechanism might be explained in part by the fact that serum cytokine concentrations in infants with RSV infection were strongly related to birth weight, not to delivery mode. IL-6 high patients had significantly worse lung function and more frequent asthma exacerbations than IL-6 low patients [21]. Circulating IL-6 is elevated in asthmatic patients and in bronchoalveolar lavage fluid of patients in whom asthma is clinically active. IL-6 levels probably reflect an activated state of the lung, and may have a role as a biomarker for asthma [22]. In the present study, increased serum IL-6 levels in infants with LBW might also play an important role in RSV induced wheezing or asthma. Although average serum IL-6 in RSV patients with wheezing was higher than in those without wheezing, there was no significant difference between them. This phenomenon further indicated that only LBW was likely to cause obvious increases in serum IL-6 levels in infants with RSV infection. In general, IL-6 is moderately increased in virus infections, lower than in *Mycoplasma pneumoniae* and bacterial infections [11, 23]. The present study also confirmed this trend. It has been previously documented that the levels of pro-inflammatory cytokines such as IL-6 were consistently higher in RSV patients at hospital admission, discharge, and 1 month after discharge than control levels. There was a trend towards greater IL-6 production in RSV infection, but not IFN-γ production [13]. These cytokines recruit inflammatory and immunocompetent cells into the sites of airway inflammation. Therefore, it is likely that the increased IL-6 response in RSV patients with wheezing would provide a substratum for the development of subsequent asthma, resulting in more significant changes related to asthma, especially in children with LBW.

Additionally, a previous study by Rusconi et al. showed that the adjusted risk ratio for development of asthma was 1.33 for elective cesarean delivery compared with spontaneous vaginal delivery [24]. However, Werner et al. found no support for the hypothesis that children delivered by caesarean section have an increased risk of asthma during the first 15–18 years of life [25]. Furthermore, delivery by caesarean section was not clearly associated with hospitalizations for asthma and other wheezing disorders up to 12 years of age compared to vaginal delivery [26]. In view of the potential role of delivery mode in the development of asthma or other wheezing diseases, we also evaluated the possible role of delivery mode in cytokine changes during infantile RSV infections. No obvious changes in serum cytokines were observed between the caesarean section and vaginal delivery groups. Therefore, the present study cannot confirm the correlation between delivery mode and cytokine changes in infantile RSV infections.

Nevertheless, our study has some limitations. Besides the six cytokines in this study, other inflammatory cytokines were reported to be related to pathogenesis of RSV infection, such as IL-5, 13, and 17 [3]. We investigated the above six cytokines only because of the availability of the commercial CBA kit consisting of the six cytokines: IL-2, 4, 6, 10, TNF-α, and IFN-γ. Secondly, because commercial respiratory immunofluorescence kit via NPA in our hospital only detected respiratory syncytial virus (RSV), influenza virus A and B, parainfluenza virus, and adenovirus, not including rhinovirus, we did not perform rhinovirus testing in the present study. It is likely that the RSV-rhinovirus co-infection is the most common co-infection, or Non-RSV infected patients might have rhinovirus infection. Thirdly, although all the samples were collected on admission, we are not certain if they were collected at the same stage of infection. Finally, this study mainly focused on a certain period of hospitalization; long term follow-up study was not performed. Therefore, larger prospective studies are necessary to explore the potential role of serum cytokines in RSV patients with LBW.

## Conclusions

In conclusion, our study demonstrated that serum IL-6 level was significantly increased in RSV infected infants with LBW, regardless of wheezing status. It is likely that specific serum cytokine patterns may contribute to the development of wheezing or asthma later in life in RSV-infected individuals, especially in RSV-infected infants with LBW.

## Abbreviation

CRP: C reactive protein
IFN: Interferon
LBW: Low birth weight
LRTI: Lower respiratory tract infection
NBW: Normal birth weight
NPA: Nasopharyngeal aspirates
PCT: Procalcitonin
RSV: Respiratory syncytial virus
TNF: Tumor necrosis factor
